# Nanoparticle‐Based Vaccines Against *Toxoplasma gondii*: Immunological Rationale, Platform Strategies, and Translational Challenges

**DOI:** 10.1155/tbed/6819201

**Published:** 2026-09-09

**Authors:** Xiaoyu Sang, Yutong Zhang, Fangqi Sun, Yanting Jin, Hua Zhang, Ying Feng, Hany M. El-Wahsh, Sara Farag, Ebtesam Al-Olayan, Beniamino Cenci Goga, Na Yang, Saeed El-Ashram

**Affiliations:** ^1^ Key Laboratory of Livestock Infectious Diseases, Ministry of Education, College of Animal Science and Veterinary Medicine, Shenyang Agricultural University, 120 Dongling Road, Shenyang 110866, China, syau.edu.cn; ^2^ Marine Biology Department, Faculty of Marine Sciences, King Abdulaziz University, Jeddah 21589, Saudi Arabia, kau.edu.sa; ^3^ Zoology Department, Faculty of Science, Kafrelsheikh University, Kafr El-Sheikh 33516, Egypt, kfs.edu.eg; ^4^ Infectious Disease Vaccines Research Chair, Department of Zoology, King Saud University, Riyadh 11545, Saudi Arabia, ksu.edu.sa; ^5^ Dipartimento di Medicina Veterinaria, Università di Perugia, Perugia 06121, Italy, unipg.it

**Keywords:** antigen presentation, lipid nanoparticles, mucosal immunity, nanoparticle vaccines, One Health, Th1 immunity, *Toxoplasma gondii*

## Abstract

*Toxoplasma gondii* is a widely distributed zoonotic pathogen with detrimental impacts on both human health and livestock production. No vaccine is available for humans, and the only licensed veterinary vaccine, Toxovax, has limitations related to safety, stability, and large‐scale production. In this review, we analyze nanoparticle platforms, including PLGA, chitosan, lipid nanoparticles, calcium phosphate nanoparticles, virus‐like particles, and porous delivery systems, in relation to antigen uptake and MHC‐I/MHC‐II presentation, Th1‐polarized cellular immunity, and mucosal vaccination. We also consider how antigen selection, carrier properties, route of administration, and host species influence immune responses and protection. Available studies show that different antigens may perform differently within the same platform and that the same antigen may induce different outcomes when delivered by different carriers. From a One Health perspective, feline vaccination may reduce environmental oocyst contamination, whereas livestock vaccination may reduce tissue cysts and food‐borne transmission. Although parenteral mRNA‐LNP vaccines have reduced chronic‐stage parasite burdens in mice, direct evidence that they induce intestinal mucosal immunity remains limited. Key translational issues include validation in nonrodent hosts, chronic‐stage and mucosal endpoints, manufacturing scalability, and regulatory approval. Nanoparticle vaccines remain promising, but their successful development will require closer matching of antigen, platform, administration route, and target host.

## 1. Introduction

There is now no approved vaccination for the human population against *Toxoplasma gondii*, which poses a worldwide health danger [1]. Besides its harmful impact on the well‐being of individuals, *T. gondii* is an economic burden for animal producers, notably in sheep and goats, because of miscarriages and infertility. Asymptomatic in most situations, toxoplasmosis can cause significant sickness in adults who are immunocompromised and in congenitally infected neonates [1, 2]. Transmitted via environmental oocysts, tissue cysts in intermediate hosts, and zoonotic infection via food and water sources, toxoplasmosis is considered a One Health problem. Th1 cells, particularly those that produce IFN‐γ, are critical in the development of immunological protection against toxoplasmosis infection [3, 4]. Studies suggest that the involvement of CD8^+^ cytotoxic T cells in the prevention of cyst reactivation and chronic infection control is crucial [5, 6]. The immune system’s response to acute tachyzoites may not be sufficient for the control of chronic bradyzoites.

Few *T. gondii* vaccines exist. Toxovax, a live‐attenuated vaccine, works in sheep, but safety, stability, and deployment difficulties limit its usage. Subunit and DNA vaccines are safer in theory, but they rarely produce strong cellular immunity and fail to deliver soluble proteins and naked nucleic acids to antigen‐presenting cells (APCs). Similarly, alum, despite being a regular component of human immunizations, largely activates Th2‐biased humoral responses and is frequently inefficient in treating intracellular parasites [7]. Delivery strategies and adjuvant approaches that boost Th1‐polarized immunity without the hazards associated with highly reactive experimental adjuvants are plainly needed.

Delivery methods based on nanoparticles provide a suitable solution to numerous restrictions at once. They can influence intracellular trafficking pathways relevant to MHC class I and class II presentation, increase antigen stability, and target delivery to APCs [8–10]. In certain cases, nanoparticle formulations—especially those containing ionizable lipid—may not only operate as delivery vehicles but also have adjuvant‐like innate immune activation. This is primarily supported by the literature on mRNA vaccines, and it is yet unclear how exactly this links to the presentation of the *T. gondii* antigen [11, 12]. These pathways are valuable for *T. gondii* because they help create the sort of immunity most closely connected to protection, in addition to improving antigen delivery.

Vaccine systems based on nanoparticles already have a solid basis in both human and veterinary medicine. The effectiveness of mRNA‐LNP vaccines against COVID‐19 has validated lipid nanoparticles as a clinically applicable platform for robust humoral and cellular immunity [13, 14]. Biocompatible polymers like PLGA have long been employed in controlled delivery systems [15, 16]. Since PLGA, chitosan, lipid nanoparticles, virus‐like particles, and related systems have demonstrated encouraging preclinical results, these developments have sparked interest in using nanotechnology in parasitic vaccinology, including *T. gondii* [17–19].

**Figure 1:**
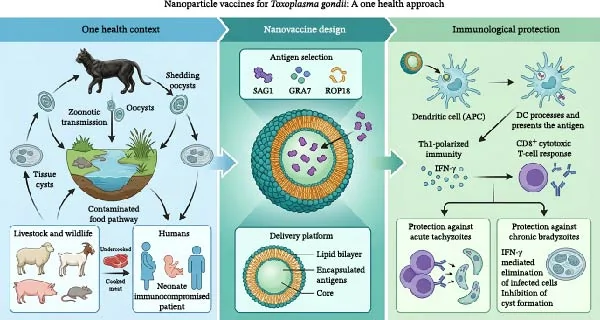
*Toxoplasma gondii* nanovaccine: One Health context and immune mechanism. Schematic depiction demonstrating the transmission life cycle of *Toxoplasma gondii* (left), design for nanoparticle vaccine administration incorporating antigen encapsulation (middle), and eliciting protective response with CD8^+^ T cells through Th1 immunity (right).

The development of nanoparticle‐based vaccine research against *T. gondii* has been addressed in recent publications [18]. Here, we supplement prior findings by emphasizing more precisely how immunological demands, antigen selection, nanoplatform selection, and translational feasibility coincide. In order to perform an organized literature survey, PubMed and Web of Science databases were searched until December 2025 using the following search terms: (“*Toxoplasma gondii*” OR “toxoplasmosis”) AND (“nanoparticle” OR “PLGA” OR “chitosan” OR “lipid nanoparticle” OR “LNP” OR “virus‐like particle” OR “VLP” OR “mRNA vaccine” OR “calcium phosphate nanoparticle”) AND (“vaccine” OR “immunization”). The selected articles for inclusion were those published as original research studies or reviews in peer‐reviewed journals only in the English language and had results related to protection or immunity from *T. gondii* infection. In a One Health design, considerable attention is made to how these dimensions interact in various application contexts. In this review, we explore vaccinations based on nanoparticles as a way to better control toxoplasmosis. We concentrate on three related questions: which immune mechanisms are most important for preventing both acute and chronic infection; how antigen design and nanoplatform selection should be combined to enhance vaccine efficacy; and how these technologies might fit into a One Health framework that includes applications for humans, livestock, cats, and wildlife. We suggest that integrating antigen selection, distribution method, and application context with the immunological requirements of protection will be more significant for advancement in this field than increasing the number of viable platforms. Figure 1 provides an overview of the organization. Its left panel places *T. gondii* transmission in the One Health context of humans, livestock, cats, and wildlife; the middle panel shows antigen selection and nanoparticle encapsulation; and the right panel links APC processing to Th1‐ and CD8^+^ T‐cell‐mediated control of acute tachyzoites and chronic bradyzoites. These three parts correspond to the immunological, platform, and translational questions discussed in the following sections.

## 2. Immunological Requirements for Protective Immunity Against *T. gondii*

### 2.1. Th1 Immunity as the Central Axis of Resistance

Resistance to toxoplasmosis relies on a persistent Th1 response, where IFN‐γ serves as an important mediator [3, 4]. IL‐12‐driven secretion of IFN‐γ is crucial both to limit parasite replication inside host cells during acute infection and to orchestrate a more complex cellular resistance. Being an obligate intracellular parasite, vaccine approaches relying solely on generating a proper environment in terms of Th1 immunity seem unlikely to confer any significant protection against *T. gondii*.

### 2.2. CD8^+^ T Cells and the Challenge of Chronic Infection

Successful immunity against *T. gondii* implies effective control of both acute and chronic parasite stages. The role of CD8^+^ T‐cell responses goes beyond effective elimination of parasites in acute infection, including their maintenance of chronic infection in its latent stage (i.e., prevention of its reactivation). As a result, evaluation of vaccine efficiency has to go beyond monitoring the amount of antibodies and immediate survival after an acute infection.

### 2.3. Why Conventional Formulations Often Underperform

One of the major challenges associated with conventional subunit vaccines relies on the relatively low capacity of soluble antigens to be effectively captured by APCs and undergo cross‐presentation to activate MHC‐I presentation [9, 20]. This explains why subunit vaccines exhibit weak immunogenic potential with respect to the activation of CD8^+^ T cells [20]. Besides, aluminum adjuvants favor stimulation of B‐cells to the expense of T‐cell response, making them inappropriate in cases when Th1‐type and CD8^+^ T‐cell responses are needed [7]. Altogether, this provides a logical explanation for the poor efficiency of most conventional vaccines against *T. gondii*.

### 2.4. Why Nanoparticle Delivery is Immunologically Relevant

**Figure 2:**
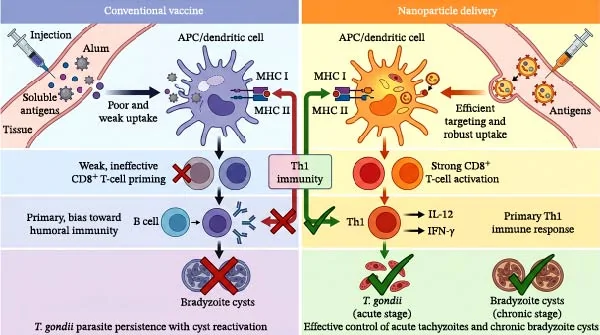
Comparison of immune responses to conventional and nanoparticle *Toxoplasma gondii* vaccines. A schematic demonstrating why nanoparticle systems outperform conventional vaccines. Conventional vaccines (left) have limited APC uptake and cross‐presentation, resulting in a humoral bias and ineffective management of chronic cysts. Nanoparticle delivery (right) enhances antigen uptake, MHC‐I cross‐presentation, and MHC‐II presentation, leading to a strong Th1/CD8^+^ axis (IFN‐γ and IL‐12) that efficiently inhibits acute tachyzoites and persistent bradyzoite cysts.

In addition to their advantages, nanoparticle delivery systems allow addressing several of those drawbacks. They provide better stability of delivered antigens while enhancing their capture by APCs and facilitating MHC‐I presentation of antigens to activate CD8^+^ T cells [8–10]. Unmodified particles are generally taken up through endocytic or phagocytic pathways influenced by particle size and surface properties [8, 10, 20], whereas ligand‐functionalized particles can promote receptor‐mediated delivery to selected APC populations [21]. APC uptake or targeting should not, however, be considered equivalent to APC maturation. In a *T. gondii* model, TgRPS2 carried by PLGA or chitosan increased the DC maturation markers CD83 and CD86. Both formulations increased MHC‐II expression, whereas MHC‐I expression was significantly increased only by the chitosan formulation [22]. An increase in MHC expression indicates greater antigen‐presentation capacity, but it does not by itself demonstrate antigen‐specific presentation, which also requires antigen processing and peptide loading onto MHC molecules [9, 23]. Antigens retained in endosomal compartments usually favor MHC‐II presentation, whereas cytosolic access and cross‐presentation are important for MHC‐I‐mediated CD8^+^ T‐cell priming [9, 20]. Some nanosystems may also influence innate immunity and cytokine balance, although most mechanistic evidence for this effect comes from general vaccine studies [11, 12]. Figure 2 illustrates this distinction by comparing conventional and nanoparticle formulations. In the left panel, limited APC uptake and cross‐presentation result in weak CD8^+^ T‐cell priming and poor control of chronic cysts. In the right panel, improved antigen uptake and intracellular processing support MHC‐I cross‐presentation, MHC‐II presentation, and a stronger Th1/CD8^+^ response. Thus, the figure emphasizes that the value of a nanocarrier depends not only on antigen uptake but also on the pathway through which APCs process the delivered antigen. Nevertheless, improved delivery cannot compensate for poorly selected antigens, making antigen choice a central issue in nanovaccine design.

## 3. Antigen Selection and Rational Design for *T. gondii* Nanovaccines

### 3.1. Antigen Choice as a Primary Design Bottleneck

Antigen choice is one of the most significant bottlenecks for nanovaccine development. In contrast to live‐attenuated vaccines, which introduce an extensive parasite proteome into the host’s immune system, subunit and nucleic acid vaccines utilize only a limited number of immunogens. Therefore, antigen selection becomes the key consideration at the very early stage of vaccine development rather than refinement at a later stage. As it is the case for many pathogens, the complexity of the *Toxoplasma* life cycle, the extensive range of tissue tropisms, and the necessity to protect against both acute tachyzoite and chronic bradyzoite infections pose additional difficulties.

### 3.2. Major Antigen Classes Under Investigation

The candidate antigens in *Toxoplasma* vaccinology were mainly selected among proteins related to the parasite’s ability to invade a cell and survive inside as well as manipulating the host cell environment. Among the antigens that are currently used in experiments are surface antigens SAG1, dense granule proteins like GRA7 and GRA12, rhoptry proteins ROP18, and microneme proteins involved in attachment and motility [19, 24, 25]. Recent experiments further supported the protective potential of SAG1, while the broader literature indicates that protection varies with the antigen format and administration route [25, 26].

### 3.3. Moving Beyond Single‐Antigen Strategies

Despite the biological significance of these proteins, single‐antigen vaccinations have not been able to completely prevent toxoplasmosis. In an attempt to bypass the immunological constraints imposed by single‐antigen vaccines, a growing body of research supports a vaccine design including multiepitopes, multivalency, and multistages [27]. The use of nanovaccines that use quadrivalent self‐amplifying RNAs within lipid nanoparticles and multiepitope peptides within PLGA nanoparticles, as well as several experiments where combined antigens provided better protection, have demonstrated this [28–30].

### 3.4. Antigen and Platform Are Interdependent

The delivery strategy and antigen selection should be taken into account jointly rather than separately. Polymeric carriers like PLGA and chitosan, which shield proteins from deterioration and promote APC uptake, work well with protein antigens such as recombinant SAG1 or GRA12. In contrast, carriers that facilitate intracellular distribution and, preferably, cytosolic release are required for DNA and mRNA constructions; lipid nanoparticles and calcium phosphate nanoparticles are especially pertinent in this regard [31, 32]. However, insufficient antigen selection is unlikely to be compensated for by better delivery. Antigen quality is at least as essential as carrier design, as demonstrated by the fact that GRA12 provided more protection than GRA7 inside the same PLGA platform [19].

### 3.5. Future Directions in Antigen Discovery

**Figure 3:**
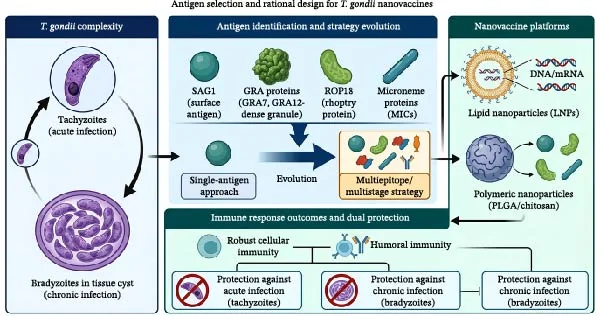
Rational antigen selection and multistage design strategies for *Toxoplasma gondii* nanovaccines. Complexity (Left): Broad‐spectrum protection is required for the tachyzoite and bradyzoite phases of parasite life. Antigen identification (top): SAG1, GRAs (7, 12), ROP18, and MICs are among the key proteins that are highlighted by selection. Strategy evolution (bottom): To increase efficacy, switch from single‐antigen methods to multivalent, and multistage formulations. Nanovaccine platforms (right): PLGA and chitosan enhance recombinant protein components, whereas LNPs are used to carry nucleic acids. Immune outcomes (bottom): Dual cellular and humoral protection against acute and chronic infection is produced by synergistic antigen‐platform combinations.

Future antigen selection should prioritize broader strain coverage and chronic‐stage relevance, both of which are currently understudied in preclinical research. More integrated strategies, such as computational epitope prediction, rational assembly of multistage antigen combinations, and reverse vaccinology, would likely be necessary to advance this field [33]. Proteins linked to invasion are still being discovered by functional studies of parasite biology and host–cell interaction, which could increase the number of potential antigens [34]. Finding antigens that are pertinent for cross‐strain coverage, platform‐compatible presentation, and chronic‐stage management is just as important as finding highly immunogenic molecules. Figure 3 brings together these considerations. It links tachyzoite and bradyzoite stages with representative SAG, GRA, ROP, and MIC antigens, shows the progression from single‐antigen to multivalent or multistage formulations, and indicates which nanoparticle systems are generally more compatible with protein or nucleic acid cargoes. The figure therefore illustrates why antigen selection and platform design should be considered jointly when protection against both acute and chronic infection is required.

## 4. Nanoparticle Platform Paradigms

Several nanovaccines based on various nanoparticle platforms were studied in *T. gondii* infection. These include polymeric (PLGA and chitosan), lipid, calcium phosphate, virus‐like, self‐assembling protein nanoparticles, and porous mucosal nanoparticle systems. The platform choice influences antigen stability, mode of delivery, APC targeting, and immune response. Table 1 provides a summary of representative studies using nanoparticles of different platforms, with cargo, predominant immune signature, and main protective effects described for each one.

**Table 1:** Representative nanoparticle‐based vaccine platforms against *T. gondii*.

Platform	Representative cargo	Key findings	Ref.
PLGA nanoparticles	GRA7/GRA12, TgRPS2, TgIMC1 DNA, and multiepitope peptides	Predominantly Th1‐biased responses; reduced parasite burden and prolonged survival in some models, with marked antigen‐dependent variation.	[19, 22, 28, 35–37]
Chitosan nanoparticles	TgRPS2, TgIMC1/P2 DNA, and SAG1 with immunomodulators	Th1 or Th1/Th17 responses; enhanced immunity and reduced parasite burden, with formulation‐dependent response patterns.	[22, 35, 36, 38, 39]
Calcium phosphate nanoparticles	GRA14 and GRA7 DNA	Enhanced Th1‐associated immunity and reduced tissue parasite loads; survival improved versus nonvaccine controls.	[31, 40]
Lipid nanoparticles/liposomes	TGGT1_316290, TGGT1_216200, quadrivalent saRNA, and GRA15 DNA	Strong humoral and cellular responses; prolonged survival and reduced parasite burden, including lower brain cyst counts with quadrivalent saRNA‐LNP.	[30, 41–43]
Virus‐like particles	Cyst wall, GRA7, and other antigens	Systemic or mucosal antibody and T‐cell responses; improved survival and reduced brain cyst burden.	[44–48]
Self‐assembling protein nanoparticles	Multiepitope constructs	Epitope‐specific CD4^+^ and CD8^+^ T‐cell responses and protection in HLA‐transgenic proof‐of‐concept models.	[49, 50]
Porous mucosal nanoparticles	Whole lysate or complex antigens	Systemic Th1/Th17‐associated responses after mucosal delivery; protection in mice and sheep.	[51, 52]
Dendrimer‐RNA nanoparticles	Multiplex self‐amplifying RNA	Humoral and cellular responses after a single dose and protection against lethal challenge.	[53]

### 4.1. Polymeric Nanoparticles: PLGA and Chitosan

PLGA remains the most extensively used platform in the development of nanovaccines against *T. gondii* due to its translational maturity and capacity to accommodate different antigen formulations [15–17]. Within *T. gondii* models, nanoformulations loaded with recombinant proteins, peptides, and even nucleic acids increase APC activation, augment IFN‐γ levels, stimulate antibody production, prolong survival, or decrease parasite load more effectively than soluble antigens or naked cargoes [19, 28, 35, 36, 54]. In addition, controlled release of the antigen can be utilized to maintain immune response and induce memory formation. However, the performance of PLGA nanoplatforms significantly depends on the antigen content. Comparison of GRA12 and GRA7 within the same nanosystem revealed more pronounced protection by the former antigen, meaning that carrier design per se does not provide reliable vaccine efficacy [19]. The coformulations with innate immune stimuli, including TLR agonists, were employed to promote the shift towards a Th1 response and improve chronic‐stage outcome [37]. On the other hand, the cationicity and mucoadhesivity of chitosan provide potential advantages as a carrier platform for mucosal delivery and nucleic acid complexation. Chitosan formulations increase DC activation, improve nucleic acid delivery, and generate a Th1‐ or Th1/Th17‐biased immune response, mainly through combination with immunomodulatory compounds, like naltrexone or propranolol [38, 39]. In plasmid vaccine experiments, chitosan nanoparticles provided increased transfection efficiency and effective parasite clearance in comparison with naked plasmid vaccines [35, 36]. Direct comparisons using the same antigen are still limited. TgRPS2 formulated in either PLGA or chitosan nanospheres increased DC maturation and T‐cell responses; PLGA produced stronger MHC‐II expression and early CD4^+^/CD8^+^ T‐cell responses, whereas chitosan was associated with stronger lymphocyte proliferation [22]. A similar comparison using pVAX1‐TgIMC1 showed that both PLGA and chitosan formulations enhanced immune responses and reduced acute‐phase parasite burdens relative to those of naked DNA. Chitosan induced stronger antigen‐specific lymphocyte proliferation and showed a more gradual release profile, whereas no clear overall carrier superiority was established [35]. Together with the GRA12/GRA7 comparison, these results indicate that particle composition, release behavior, intracellular trafficking, and antigen‐carrier compatibility may all affect protection. Because most of these factors were not examined independently, the proposed mechanisms should be interpreted cautiously. The available direct comparisons are summarized in Table 2.

**Table 2:** Direct comparisons of antigen and carrier effects in *T. gondii* nanovaccine studies.

Comparison	Main finding	Key limitation	Ref.
PLGA‐GRA12 vs. PLGA‐GRA7	PLGA‐GRA12 produced stronger immune responses, lower parasite burdens, and longer survival.	Particle size and release profiles differed, so the antigen effect was not fully isolated.	[19]
TgRPS2‐PLGA vs. TgRPS2‐chitosan	Both enhanced immunity and reduced parasite burden; no clear overall carrier advantage was established.	Immune response patterns differed and chronic protection was not tested.	[22]
pVAX1‐TgIMC1/PLGA vs. pVAX1‐TgIMC1/chitosan	Both enhanced immune responses and reduced cardiac parasite burden.	Loading and release differed and clear carrier superiority was not demonstrated.	[35]

### 4.2. Nucleic Acid Delivery Platforms: Lipid Nanoparticles and Calcium Phosphate Nanoparticles

The lipid nanoparticle route is also among the leading methods for developing nucleic acid‐based vaccines against *T. gondii* as it enables the expression of an endogenously produced antigen and cellular immunity activation [13, 14]. The lipid nanoparticles ensure the stability of labile RNA, increase its cellular uptake, and allow its release inside the cells, facilitating the synthesis of endogenously produced antigens and their presentation via MHC class I molecules [32, 55]. Indeed, mice immunized with mRNA‐LNPs carrying antigens, such as TGGT1_316290 and TGGT1_216200, survived better and had increased cellular immunity in the *T. gondii* infection model [41, 42]. Finally, in recent studies, quadrivalent saRNA‐LNP vaccines have improved this approach by including the delivery of antigens from multiple stages and protecting in both acute and chronic settings [30]. Lipid nanoparticle‐based delivery has been considered for more complex mRNA vaccines but also for simple liposome‐formulated plasmid DNAs, such as ssPalmE‐LNP TgGRA15, which is protective in mice [43].

It is possible that ionizable lipids affect immune programming. Indeed, based on the literature on mRNA vaccines, it has been established that the use of ionizable lipids can affect the signaling via innate receptors and thus can impact further adaptive response formation, namely, cytokine production, activation of APCs, and T‐cell priming [11, 12, 56]. It is yet unclear whether these effects are important specifically for the presentation or cross‐presentation of *T. gondii* antigens; thus, any mechanistic extrapolation is to be applied cautiously.

Most *T. gondii* mRNA‐LNP studies have used intramuscular immunization and have mainly measured systemic antibody and splenic T‐cell responses [41, 42]. The quadrivalent saRNA‐LNP vaccine also reduced the brain cyst burden by 72.5% after an intraperitoneal oocyst challenge [30]. This finding shows that parenteral vaccination can provide protection after intraperitoneal exposure, but it does not demonstrate that intestinal mucosal immunity was directly induced. Intestinal secretory IgA, mucosal tissue‐resident T cells, and local cytokine responses were not assessed in these studies. The capacity of injected mRNA‐LNP vaccines to generate robust intestinal immunity therefore remains uncertain, and direct comparisons of intramuscular, intranasal, and oral delivery using mucosal endpoints are needed.

Calcium phosphate nanoparticles present an alternative inorganic method for plasmid DNA delivery. Specifically, in *T. gondii* models, calcium phosphate nanoparticles have been used to deliver plasmids encoding GRA14 and GRA7, producing stronger Th1‐associated responses, favorable IgG‐subclass profiles, and reduced tissue parasite loads compared with naked‐DNA controls [31, 40]. Although calcium phosphate nanoparticles are used less frequently than LNPs in current vaccinology, their biocompatibility and ease of preparation make them a useful alternative.

### 4.3. VLPs, SAPNs, and Structured Antigen‐Display Systems

VLPs and SAPNs combine particulate structures with antigen display on their surfaces. The repetitive architecture may increase interaction with B‐cells and promote the activation of CD4^+^ and CD8^+^ T cells, depending on the design [57, 58]. Protection against acute and chronic *T. gondii* infection has been demonstrated using VLPs, including lower brain cyst burden and a better survival rate [44–48].

SAPN‐based systems deliver defined T‐cell epitopes along with helper signals and additional innate immune stimulation provided by molecules like flagellin. This approach provides a model of precise antigen display and has generated CD4^+^ and CD8^+^ T‐cell responses in genetically modified mice [49, 50]. However, the translational readiness of SAPN‐based systems remains lower than that of more established platforms such as PLGA or LNPs.

Delivery of RNA via dendrimer systems represents a novel approach to RNA vaccine development. These studies show that self‐amplifying RNA vaccines with built‐in innate immune stimulation can provide strong protection in proof‐of‐concept models [53]. At present, however, dendrimer‐RNA systems should be regarded primarily as tools for RNA vaccine design rather than as translation‐ready platforms for toxoplasmosis vaccines.

### 4.4. Mucosal and Porous Nanoparticle Systems

Due to the main infection routes, mucosal immunity remains important for preventing toxoplasmosis in veterinary applications. Porous maltodextrin‐based nanoparticle systems delivering complex parasitic antigens were shown to work effectively in mice and sheep models, protecting animals from latent and congenital infection [18, 51, 52]. Importantly, these studies demonstrate that it is possible to go beyond classical lethal mouse models and move towards animal vaccination studies.

### 4.5. Comparative Platform Perspective

Each nanoparticle platform is more appropriate for one or another vaccine goal, and there is no platform that could be regarded as universally superior for *T. gondii*. The selection is contingent on the cargo type, required immune response, administration route, host species, and delivery specifics. Proteins are most suitable to be delivered via PLGA, chitosan, and VLPs. On the other hand, nucleic acid vaccines require carriers like liposomes or calcium phosphate nanoparticles that deliver cargo into the cells where the antigen is expressed in situ [31, 32, 40]. Chitosan nanoparticles may offer advantages for mucosal delivery because of their mucoadhesive properties [38, 55]. Direct intranasal protection against toxoplasmosis has been demonstrated more clearly with porous maltodextrin nanoparticles in sheep [52].

One problem with the current literature on the subject is that it discusses the efficiency of various platforms without considering the specific experimental conditions under which it occurs. In reality, any distinctions in the performance of one platform compared to another are difficult to disentangle from the antigen used, the route of administration, the challenge, and the outcome measured. Survival after acute infection, the load of parasites in organs, chronic cyst management, and preventing transmission are all interrelated endpoints but are not interchangeable. The issue, therefore, is not which platform works better in general but which platform is more appropriate for a particular antigen, target, species, and application scenario. Table 2 summarizes the limited within‐study comparisons in which either the antigen or the carrier was held constant. These comparisons make the effects of antigen choice and particle composition easier to distinguish while also showing that the proposed mechanisms have not yet been tested independently.

## 5. Preclinical Efficacy and Translational Outlook in a One Health Context

### 5.1. Preclinical Findings

In animal models, most nanovaccines target *T. gondii* induce a Th1‐biased response. Common observations include Th1‐associated cellular and antibody responses, APC activation, and lymphocyte proliferation [19, 22, 38, 40]. Reported beneficial results have included extended survival following acute infection, a decreased parasite load within tissues, and, in some cases, a lower cerebral cyst count. The current state of evidence in the preclinical domain is highly heterogeneous. Divergent choices of antigen, mode of delivery, strain, dosage schedule, and measurement endpoints abound. A significant portion of preclinical literature continues to depend upon RH strain survival assays for validation, but recent studies have started to employ chronic‐stage measurements like the parasite cyst count [30]. Potential variations in platform efficacy must thus be considered in light of their possible contribution from factors, such as antigen selection and experimental design.

### 5.2. Livestock Vaccination and Food Safety

*Toxoplasma* infection from contaminated meat is the major route of human toxoplasmosis. From a One Health perspective, the vaccination of livestock appears to be a feasible measure. A reduction in tissue cysts will be advantageous for animal health, food safety, and public health [59, 60]. As for nanoparticle‐based strategies, the intranasal vaccination of sheep using porous maltodextrin nanoparticles has been shown to be effective in protecting animals from latent and congenital toxoplasmosis [52]. Thus, it appears that some of the most pertinent technologies are those that are closest to actual requirements rather than those that have the greatest mouse immunogenicity.

### 5.3. Feline Vaccination and Interruption of Environmental Transmission

Cats are the definitive hosts and carriers of environmentally stable oocysts. It might be possible to interfere with the transmission chain at its source by decreasing oocyst shedding with the help of vaccination. There are feline‐oriented vaccines developed already; however, they are not used in clinical practice yet [5]. The experience gained in such areas as research on rROP2 or genetically attenuated strains demonstrates that such an approach is feasible [61, 62]. In cats immunized with the live‐attenuated RHΔ*ompdc*Δ*uprt* strain, oocyst shedding was reduced by 95.3% after challenge [61]. Vaccination with recombinant ROP2 also reduced oocyst shedding, although the degree of protection depended on the immunization protocol [62]. These are not nanoparticle vaccines, but they establish the reduction of oocyst shedding as a relevant endpoint for feline vaccine development. Because cats are the source of environmentally resistant oocysts, even partial control of shedding may reduce the exposure of livestock, wildlife, and humans.

### 5.4. Wildlife Conservation and Human Translation

*T. gondii* has an effect on species, particularly New World primates and marine creatures, linking environmental contamination to biodiversity loss [63, 64]. Nanoparticle‐based immunization has shown promise in sensitive confined animals, indicating a possible role in conservation medicine [65]. For human use, the translation procedure is more complex. Priority categories could include seronegative women before pregnancy and immunocompromised patients who are at high risk of serious disease. Clinical and regulatory experience with PLGA and mRNA‐LNP vaccines provides platform‐level precedent, but it does not guarantee safety or efficacy in *T. gondii*‐specific applications. These questions remain unsolved and will demand more appropriate preclinical and translational models.

### 5.5. Major Translational Barriers

**Figure 4:**
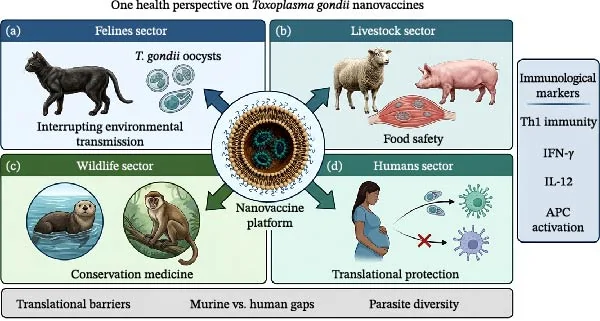
Host‐tailored One Health framework for *Toxoplasma gondii* nanovaccine translation. The central element represents a host‐tailored design process rather than a single universal formulation and connects four application areas: (a) Felines, interrupting environmental transmission by reducing oocyst shedding; (b) livestock, enhancing food safety by limiting tissue cyst formation in sheep and pigs; (c) wildlife, supporting conservation medicine in susceptible species; and (d) humans, addressing high‐risk populations, including seronegative women before pregnancy and immunocompromised patients. Successful deployment requires overcoming major translational barriers, including species‐dependent immune responses, murine–human immunological discrepancies, parasite genetic diversity, formulation limitations, and the lack of standardized cross‐species validation.

One key difficulty is the distance between mouse models and human applications. Mice use TLR11/TLR12‐dependent recognition of *T. gondii* profilin, whereas this pathway is not functionally conserved in humans [5, 66]. This difference should be considered when extrapolating murine vaccine responses to humans. In the later stages of preclinical testing, humanized models and primary human immune cells will be important to close this gap. Another problem is parasite diversity. Natural *T. gondii* populations are genetically varied, but most research still relies on a small number of laboratory strains, principally RH and ME49 [67]. This limits confidence in broader applicability and underscores the importance of cross‐strain protection. Formulation limitations are also significant. mRNA‐LNP technologies may face formulation, storage, and implementation challenges [68]. Polymeric or mucosal platforms could offer practical advantages in some veterinary or field settings, although this possibility requires a direct comparative evaluation. Future development should be guided by target product profiles, not just murine immunogenicity (Figure 4).

Direct testing of the same nanoparticle vaccine across several host species remains uncommon. Porous maltodextrin formulations have progressed from mouse studies to sheep vaccination [51, 52], and nanoparticle‐based antigens have also been evaluated in susceptible squirrel monkeys [65], but these studies were not standardized head‐to‐head comparisons. The vaccination objective should be adjusted to the host: reduction of oocyst shedding is the principal endpoint in cats; reduction of tissue cysts and congenital infection is more relevant in livestock; oral or mucosal delivery and field stability are important for wildlife; and human candidates require formulations that account for the absence of functional TLR11/TLR12 pathways and meet a higher safety threshold [5, 52, 63, 65, 66]. Figure 4 integrates these host‐specific priorities within a One Health framework. Rather than proposing a single formulation for all hosts, the figure links the nanovaccine design to different control objectives. It also highlights the shared barriers between murine proof‐of‐concept studies and practical application, including species‐dependent immune responses, parasite genetic diversity, formulation limitations, and the lack of standardized cross‐species validation. Thus, the antigen, nanoparticle platform, administration route, and efficacy endpoint should be selected according to the intended host and application setting.

## 6. Conclusion and Future Perspectives

In addition to addressing challenges related to adjuvants, nanovaccines represent an attractive approach for solving two important problems with *T. gondii* vaccines: weak cellular immunity induced by soluble antigens and the lack of safe formulations suited to an intracellular parasite. While promising results are already obtained, the field requires fewer proof‐of‐concept experiments and more focused comparative analysis with mechanistic understanding aimed at vaccine translation.

A number of factors will play a key role in deciding whether the recent progress in nanotechnology leads to viable vaccines against Toxoplasma. One is the difference between murine and human innate immunity [5, 66]. Another is the narrow range of laboratory strains, such as RH and ME49, used in most studies and the resulting difficulty of extending findings to genetically diverse parasite populations [67]. Finally, mRNA‐LNPs are promising but still require substantial formulation optimization before widespread clinical application [68].

There are several promising avenues of research that may result in new advances in nanotechnology being applied to *T. gondii* vaccines. As a conceptual future strategy, hybrid nanoparticles combining lipid and polymeric components may offer both sustained release and intracellular delivery, although this approach has not yet been directly validated for *T. gondii* vaccines [55, 69]. Machine learning techniques can facilitate the search for multistage, cross‐reactive antigens. In general, further research should focus on standardized chronic‐stage assays, cross‐strain challenge models, nonmurine validation, and direct comparison of different antigen‐nanoparticle combinations under identical conditions. From the One Health perspective, the best chances of achieving tangible results are associated with the vaccination of livestock and cats.

## Funding

This research was funded by grants from the National Natural Science Foundation of China (Grants 32072891, 32373039, 31672546, and 31902297), the Liaoning Provincial Science and Technology Joint Program (Grant 2025‐MSLH‐610), the Ongoing Research Funding Program‐Research Chairs (Grant ORF‐RC‐2026‐4500), and the King Saud University, Riyadh, Saudi Arabia.

## Conflicts of Interest

The authors declare no conflicts of interest.

## Data Availability

Data sharing is not applicable to this article as no datasets were generated or analyzed during the current study.
